# A Novel Laser 3D Printing Method for the Advanced Manufacturing of Protonic Ceramics

**DOI:** 10.3390/membranes10050098

**Published:** 2020-05-12

**Authors:** Shenglong Mu, Yuzhe Hong, Hua Huang, Akihiro Ishii, Jincheng Lei, Yang Song, Yanjun Li, Kyle S. Brinkman, Fei Peng, Hai Xiao, Jianhua Tong

**Affiliations:** 1 Department of Materials Science and Engineering, Clemson University, Clemson, SC 29634, USA; smu@g.clemson.edu (S.M.); yuzheh@g.clemson.edu (Y.H.); hhuang8@clemson.edu (H.H.); aishii@clemson.edu (A.I.); ksbrink@clemson.edu (K.S.B.); fpeng@g.clemson.edu (F.P.); 2 Department of Electrical and Computer Engineering, Clemson University, Clemson, SC 29634, USA; jinchel@g.clemson.edu (J.L.); song5@g.clemson.edu (Y.S.); yanjun@clemson.edu (Y.L.); haix@clemson.edu (H.X.)

**Keywords:** protonic ceramics, 3D printing, laser processing, fuel cells, membrane reactors

## Abstract

Protonic ceramics (PCs) with high proton conductivity at intermediate temperatures (300–600 °C) have attracted many applications in energy conversion and storage devices such as PC fuel/electrolysis cells, PC membrane reactors, hydrogen pump, hydrogen or water-permeable membranes, and gas sensors. One of the essential steps for fulfilling the practical utilization of these intermediate-temperature PC energy devices is the successful development of advanced manufacturing methods for cost-effectively and rapidly fabricating them with high energy density and efficiency in a customized demand. In this work, we developed a new laser 3D printing (L3DP) technique by integrating digital microextrusion-based 3D printing and precise and rapid laser processing (sintering, drying, cutting, and polishing), which showed the capability of manufacturing PCs with desired complex geometries, crystal structures, and microstructures. The L3DP method allowed the fabrication of PC parts such as pellets, cylinders, cones, films, straight/lobed tubes with sealed endings, microchannel membranes, and half cells for assembling PC energy devices. The preliminary measurement of the L3DP electrolyte film showed a high proton conductivity of ≈7 × 10^−3^ S/cm. This L3DP technique not only demonstrated the potential to bring the PCs into practical use but also made it possible for the rapid direct digital manufacturing of ceramic-based devices.

## 1. Introduction

In recent decades, along with the development of new materials for achieving high proton conductivity [1,2,3], protonic ceramics (PCs) have achieved significant progress on intermediate-temperature (300–600 °C) protonic ceramic energy devices (PCEDs) such as PC fuel cells [4,5,6,7,8], PC electrolysis cells [9], reversible PC electrochemical cells [10,11,12,13], PC membrane reactors [14,15,16], hydrogen-permeable membranes [17,18], water-permeable membranes, and solid-state ammonia synthesis cells [19,20,21,22,23]. However, so far, most of the promising results for these PCEDs relied on the small cells (≤1.0 cm^2^) with a simple planar geometry [6] fabricated by the traditional dry pressing or screen printing methods. The scalable tape casting method has shown the potential to manufacture large-area PC fuel cells (e.g., ≈16 cm^2^), which, however, has to face the delamination problem and limitation of the simple planar geometry [24,25,26]. The paste extrusion method has proven the success of the fabrication of tubular PC fuel cells and PC membrane reactors with large active areas (e.g., ≈20 cm^2^), which, however, can do nothing for the fabrication of the convoluted tubes with a closed-end or with surface lobes for increasing surface-area-to-volume ratio [27,28]. The existing challenges of the traditional ceramic manufacturing techniques have become the significant obstacles for the practical application of PCEDs because it is almost impossible to cost-effectively and rapidly manufacture sizable PCEDs with a large active area, high surface-area-to-volume ratio, controllable microstructures, and desired performance in a customized on-demand way.

The new advanced manufacturing technique of additive manufacturing (i.e., 3D printing) fabrication process starts from designing 3D models of the objects by computer-aided design (CAD) software and then slices the models to successive cross-sectional layers. After that, the 3D printing machines deposit these slices together to build the parts in a layer-by-layer way. We can say that 3D printing is a process of *What You See Is What You Build*, which is particularly valuable for complex geometries. Recently, the 3D printing technologies have expanded their applications from rapid prototyping to direct digital manufacturing of final products with desired properties and functions. The 3D printing technique has been widely applied to the fields of aerospace, automotive, biomedical, consumer goods, and others [29] since its unique process is suitable for the fabrication of the parts/devices having complicated outside geometries and inside complexity [29,30]. However, most of the commercially available 3D printers are dedicated to the manufacturing of polymers and metals/metal alloys [31,32] because of the easiness of consolidation or sintering of these materials. Although the 3D printing of ceramics has caught significant attention too, the common challenges for ceramic processing such as the difficulty for achieving high accuracy due to the significant shrinkage, the difficulty for fulfilling crack-free rapid sintering due to the intrinsic brittleness, the difficulty for depositing precise layers due to the heavy involvement of additive materials (e.g., polymer binders and solvents) are the significant obstacles for the 3D printing-based advanced manufacturing. 

In this study, we developed a new L3DP advanced manufacturing process by integrating digital microextrusion-based 3D printing and rapid and precise laser processing (drying, sintering, cutting, and polishing). We showed that the L3DP method is capable of manufacturing PCs with desired complex geometries, crystal structures, and microstructures. We further demonstrated the success of manufacturing PC parts such as pellets, cylinders, cones, films, straight/lobed tubes with sealed endings, microchannel membranes, and half cells for assembling PCEDs. The preliminary characterization of the L3DP-derived PC electrolyte films showed a high proton conductivity, which provided a substantial prerequisite for the successful PCEDs.

## 2. Experimental Section

### 2.1. L3DP System

**Figure 1:**
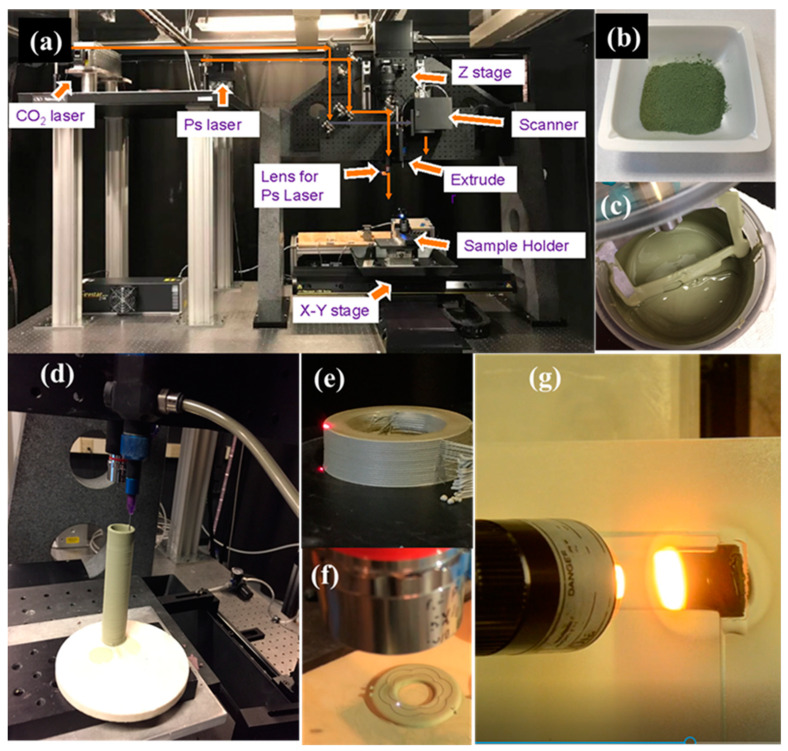
Our in-house L3DP system for the advanced manufacturing of protonic ceramic energy devices (PCEDs). (**a**) Photo of L3DP system, (**b**) ball-milled precursor powder, (**c**) preparation of printable paste, (**d**) 3D printing based on microextrusion, (**e**) rapid laser drying during 3D printing, (**f**) rapid laser machining during 3D printing, and (**g**) rapid laser sintering of green layers.

Figure 1a provides a photo of our in-house advanced manufacturing system of L3DP. The L3DP system consists of X-Y and Z stages, microextruders (preeflow eco-PEN 300, ViscoTec, Töging am Inn, Germany), a CO_2_ laser (firestar v20, wavelength 10.64 μm, SYNRAD, Inc., Mukilteo, WA, USA), a picosecond YAG laser (hereafter ps-laser, wavelength 1064 nm, Olive-1064-10, Attodyne, Inc. Toronto, Canada), and a Galvano scanner (intelliSCAN® III 14 (ID# 128650)). The X-Y stage can move with very high precision (100 nm) with a wide speed range (from 100 nm/s up to 5 m/s). The CO_2_ laser was used for rapid drying and rapid sintering. The ps-laser was used for precise polishing and cutting. The Galvano scanner was used to provide the faster movement of lasers for drying, sintering, polishing, and cutting. The L3DP system allows us to do advanced manufacturing of green or sintered ceramic parts easily by combining the 3D printing based on fast microextrusion, accurate subtractive manufacturing based on laser processing, and in-situ consolidation based on high-energy laser sintering. Starting from paste preparation from ball-milled precursor powders [33], by using the L3DP system, we can do the microextrusion-based 3D printing [34], rapid laser drying, rapid sintering, precise laser polishing, and precise laser cutting for the advanced manufacturing of PC parts (Figure 1b–g). The details of each experimental operation are described in the following experimental sections.

#### 2.1.1. Preparation of Printable Pastes

Using the 40 wt% BaCe_0.7_Zr_0.1_Y_0.1_Yb_0.1_O_3-δ_ (BCZYYb) + 60 wt% NiO hydrogen electrode material as an example, we prepared the pastes of the PC parts as the following procedures. The stoichiometric amounts of carbonate and oxide precursors (i.e., BaCO_3_, CeO_2_, Y_2_O_3_, ZrO_2_, Yb_2_O_3_, and NiO) got ball-milled for 48 h with isopropanol as grinding solvent and 3 mm YSZ as grinding media. Then the dry ball-milled powder (Figure 1b) was mixed with 15 wt% of deionized water, 0.7 wt% of dispersant (Darven 821A, Vanderbilt Minerals, CT, USA), and 1–3 wt% (based on water amount) of binder HPMC (hydroxypropyl-methylcellulose, Alfa Aesar, MA, USA) by a vacuum mixer (VPM MINI COMP W/STAND 115V) for 30 min. The printable pastes (Figure 1c) were used for the fabrication of 2D and 3D PC parts. The pastes for the manufacturing of other PC parts with different compositions were also prepared using the same procedures. The amounts of water, dispersant, and binder might be adjusted to some degree according to the differences in material compositions. 

#### 2.1.2. Microextrusion-Based 3D Printing 

The different kinds of fresh pastes prepared in Section 2.1.1 were fed to the pre-designated plastic syringe reservoirs, respectively, to avoid the mixed contamination. The compressed air was applied to drive the paste to the microextruder with a needle-type nozzle of 0.5 mm in diameter (Figure 1d). The distance *h* between the extruder nozzle and platform substrate usually is equal to the thickness of the wet layer (the printed layer before drying) introduced by 3D printing, which is around 450 μm for most of the 3D printing in this work. Usually, the paste extrusion flow rate *Q* is 0.3 mL/min, and the stage moving speed *v* is 15 mm/s. Under these conditions, a filament with a width *d* of ≈740 μm can be obtained according to the following, Equation (1) [34]:

(1) $$d=\frac{Q}{vh}$$

The tool paths for printing each layer vary versus the different geometries of the PC parts. Usually, the printing tool path could be adjusted to satisfy the requirement of the desired part geometry. For example, a tubular part was printed using a spiral line path, while a simple square thin film was printed using a line-by-line bi-directional path. 

#### 2.1.3. Rapid Laser Drying

The pastes sometimes contained a small additional amount of solvent to allow the smooth printing and effective bonding between the fresh layer and the previous layer. However, the natural drying of the low-viscous layer in ambient air took so long that it not only significantly slowed down the 3D printing process but also caused the shape deformation due to paste gravity and fluidity. In our L3DP system, the CO_2_ laser was used to dry each wet green layer (Figure 1e) just after printing for speeding up the 3D printing process and avoiding the shape deformation. The laser beam was usually defocused by 15 mm to increase its spot size to ≈1 mm and lower the laser energy density for avoiding overheating. The optimized laser operation parameters of laser power of ≈10 W and a scan rate of 15 mm/s were able to efficiently dry the green layers without noticeable shrinkage and reactions observed. To further decrease the time consumption for drying, the Galvano scanner was equipped with the CO_2_ laser, which allowed use of the higher laser power and the faster scan rates for the drying process. 

#### 2.1.4. Precise Laser Machining

The laser machining was performed using the ps-laser equipped with the L3DP system (Figure 1f). The cutting and polishing operations on the green layers were studied. For laser cutting, the ps-laser was focused on a spot with a size of 18 μm using a 5× lens (NA = 0.13). The repetition rate, laser energy, and laser scan rate were 10 kHz, 150 μJ per pulse, and 5 mm/s, respectively. Under these conditions, the ps-laser usually could cut a depth of 150 μm in the green layers. The small unit cutting dimension of 18 × 150 μm usually could result in very accurate cutting, which allowed to cut microchannels to make highly compacted microchannel membrane reactors or cutting edges and complicated contours to make complicated geometries for achieving larger area to volume ratio and improve the 3D printing feature accuracy [35,36]. By setting the proper laser operation parameters, the ps-laser could also be used to polish the surface of 3D-printed green layers or parts to allow the achievement of smooth finishing surface to next-step processing (e.g., dip-coating) [25]. In this experiment, the laser operation parameters of the repetition rate of 1 kHz, the laser energy of 114.4 μJ per pulse, and the laser scan rate of 50 mm/s were used for polishing green layers or parts obtained by the microextrusion-based 3D printing. 

#### 2.1.5. Rapid Laser Reactive Sintering

Based on the solid state reactive sintering (SSRS) developed by Tong et al., we developed rapid laser reactive sintering (RLRS) for rapid sintering PCs [33,37,38]. For example, the thoroughly dried BCZYYb electrolyte green layer printed on the fused silica substrate was subjected to the RLRS [33]. The green component can be sintered into the desired crystal structures and desired microstructures. The CO_2_ laser fixed on Z-axil (λ = 10.6 µm, Ti100W, Synrad) was applied for the RLRS of the PC parts (Figure 1g). In this work, the CO_2_ laser through a cylindrical lens formed a line shape laser spot and was applied for the sintering and densifying the electrolyte layers. The laser beam with a defocus distance of 20 mm, and a beam spot size of around 8 mm was used for achieving homogenous and moderate laser energy density. The laser power and the laser moving speed were set to be 20 W and 0.1 mm/s, respectively. The microstructures of the sintered layers were controlled by optimizing the laser parameters such as laser power, moving speed, defocus distance, and space of the hatching pattern.

### 2.2. Post Treatment

The L3DP method involving laser sintering could obtain sintered PC parts with desired microstructures, crystal structures, and geometries, which could directly be subjected to the measurement for properties or be assembled for constructing devices. However, in most cases, the L3DP method resulted in the PC green parts, which need proper post-treatments such as coating and sintering to make the PC parts with desired microstructures, properties, and functions. The following post-treatments were performed to obtain the tubular PC half-cells from the L3DP-derived 40 wt% (BaZr_0.8_Y_0.2_O_3-δ_) BZY20 + 60 wt% NiO anode support. The L3DP-derived green anode tubes were first prefired at 1050 °C for 12 h using a conventional box furnace to thoroughly vaporize paste solvent and partially burn the binders or dispersant. After that, the BZY20 + 1 wt% NiO electrolyte precursor slurry comprised of 50 wt% electrolyte precursor powder, 2 wt% of dispersant (Polyethylene glycol), 5 wt% of the binder (Heraeus V006), and 43 wt% of ethanol solvent was dip-coated on the outside surface of the tubes. After the dip coating, the green half-cells were dried in air for two days. Finally, the co-firing of the green half-cells comprised of 40 wt% BZY20 + 60 wt% NiO anode and BZY20 + 1 wt% NiO electrolyte was carried out at 1450 °C for 12 h with a ramp rate of 1 °C/min. As for the single-component PC green parts such as pellets, cylinders, cones, and even microchannel-embedded green membranes, the conventional firing/sintering at high temperatures was performed in a box furnace to obtain the sintered PC parts. 

### 2.3. Characterization

The viscosity of the pastes was quantified using a rotational viscometer (ViscoLead Adv R, Fungilab, Barcelona, Spain). The paste was added into the container with an R4 spindle for measurement at room temperature. The relative density (porosity) of some representative sintered samples was measured using the Archimedes method. The microstructure of the L3DP-derived PC parts was characterized using a scanning electron microscope (SEM, Hitachi S4800, Hitachi, Ltd., Tokyo, Japan). The SEM images were mostly taken at 20 KV and 20 μA, with magnifications at 500, 1k, and 2.5k. The crystal structures of the sintered PC samples were characterized using XRD (Rigaku Ultima IV, Cu-Kα) with angle range 15–85° at 1°/min scan speed. The conductivity of the RLRS-BCZYYb electrolyte film was measured using a Gamry Reference 3000 at different atmospheres (wet air and 5% hydrogen) and temperatures. The gas flow rate was 20 ml/min, and the temperature ramp rate was 2 °C/min. 

## 3. Results and Discussion

### 3.1. Printable Pastes 

**Figure 2:**
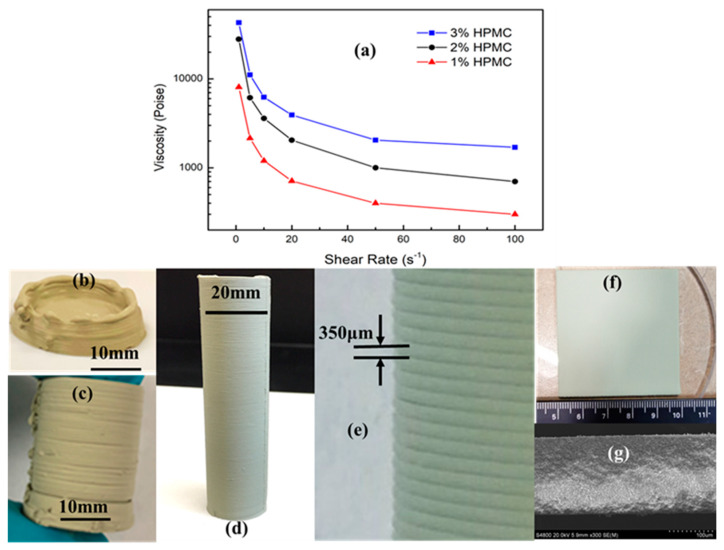
Viscosity vs. shear rate for 40 wt% BZY20 + 60 wt% NiO anode precursor pastes prepared by adding different amounts of binder (HPMC—hydroxypropyl-methylcellulose) (**a**) and effect of paste viscosity on the 3D printing performance. (**b**–**d**) The photos of the 40 wt% BZY20 + 60 wt% NiO anode green tubes 3D printed from pastes with 1, 3, and 2 wt% of HPMC binder, (**e**) the high-magnification photo of 3D printed high-quality tube shown in (d). (**f**) The photo of a 40 wt% BCZYYb + 60 wt% NiO anode green film 3D printed from paste with 1 wt% HPMC binder and (**g**) the SEM image of this film’s cross-section.

The viscosity of the pastes is one of the most critical parameters to control the quality of microextrusion-based 3D printed parts because it directly determines the shape retention of the as-printed filaments and the bonding between different layers [39,40]. In general, the increase in the paste viscosity can improve the shape retention property, while the adhesion (bonding) between layers becomes weak, which usually results in the high probability of the delamination. Therefore, the viscosity of the paste has to be optimized to meet the desired requirements for the microextrusion-based 3D printing. Although the ceramic powder to water (C/W) ratio and the amount of binder both can adjust the viscosity of the paste to the desired range, in this work, we fixed the C/W ratio at 1:1 to prevent cracking during drying and varied the amount of the binder to optimize the paste viscosity. The 40 wt% BZY20 + 60 wt% NiO precursor pastes of the PC fuel cell anode were prepared with 1, 2, or 3 wt% HPMC binder. The viscosity versus the shear rate for these three pastes was investigated and shown in Figure 2a. All three pastes showed decreasing viscosity sharply when the shear rate initially increased from zero to ≈20 s^−1^. With the further increase in the shear rate, the viscosity leveled off quickly for all the pastes. The steady viscosities of ≈100, ≈700, and 1700 P were obtained for the three pastes with 1, 2, and 3 wt% HPMC binder, respectively. 

The photos of the green parts manufactured by 3D printing from these three pastes are shown in Figure 2b–f. It can be clearly seen from Figure 2b that the paste with a low viscosity of ≈100 P prepared by adding 1 wt% HPMC could not sustain the paste weight when printing the thin wall parts (e.g., tubes). The printed paste filament flattened and changed shape quickly before it was dried naturally in the ambient environment. However, the paste with a low viscosity was suitable for the printing of 2D objects such as thin films. Figure 2f shows that the smooth and homogenous green thin films with area ≈25 cm^2^ were successfully printed from this low viscous paste. The use of low viscous paste in microextrusion-based 3D printing provided an effective way to achieve thin and uniform layers because of the flattening of individual paste filaments and the merging between neighboring filaments. The SEM image of the thin film (Figure 2g) shows that the uniform green film can be as thin as 180 μm. Figure 2c indicates that the paste with high viscosity of ≈1700 P (3 wt% HPMC) resulted in a weak bonding between the previous filament and the fresh filament. Although the shape retention was kept well, the evident delamination was observed in the 3D printed green tubes. The medium viscous paste with 2wt% HPMC allowed the successful 3D printing of thin-wall green parts. Figure 2d indicates that the 3D printed green tubes not only obtained the desired shape without deformation but also achieved excellent bonding between layers or filaments. The high-magnification image (Figure 2e) of this tube shows that the layer thickness (≈350 μm) and the connection valley between layers both are very homogenous. 

### 3.2. 3D Printing of Complex PCED Green Anode Parts

**Figure 3:**
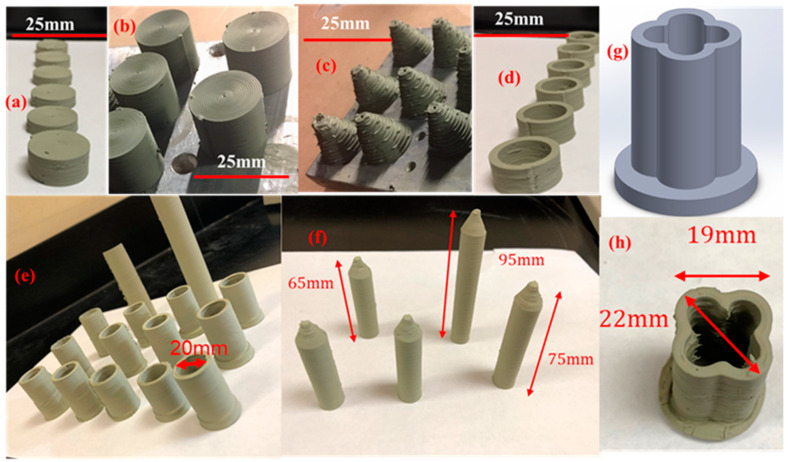
Protonic ceramic (PC) fuel cell anode 40 wt% BZY20 + 60 wt% NiO green parts prepared from direct microextrusion-based 3D printing. (**a**) Pellets, (**b**) cylinders, (**c**) cones, (**d**) rings, (**e**) straight tube with a closed bottom, (**f**) straight tubes with a closed top, and computer-aided design (CAD) 3D model (**g**) and printed (**h**) lobed-tube with a closed bottom.

The microextrusion-based 3D printing without any consolidation treatment was investigated to manufacture the PC green parts with the PC fuel cell anode of 40 wt% BZY20 + 60 wt% NiO as a case study. The green parts of pellets, cylinders, cones, rings, bottom-closed straight tubes, top-closed straight tubes, and top-closed and lobed tubes were manufactured by microextrusion-based 3D printing. The photos of these parts are summarized in Figure 3. The PC pellets are commonly used for the characterization of microstructure and properties, which are also one of the most accessible parts to be manufactured by 3D printing. Figure 3a provides the photos of six green pellets with a diameter of ≈20 mm and thicknesses of 10–20 mm. Both the top view and the side view indicate that the pellets have enough smoothness and homogeneity for further investigation. Figure 3b indicates that the solid cylinders with the same diameter (≈20 mm) as the pellets but a height of 30 mm could be easily manufactured by 3D printing, which again shows excellent smoothness and homogeneity. The PC fuel cell anode cylinders allow us to make bars for properties measurement (e.g., cut into rectangular bars for four-probe DC electrical measurement) or other long-dimension parts for further processing (e.g., produce single crystals using sintered long cylinders). The 3D printing of both pellets and cylinders only needs to print the same cross-section of green layers repeatedly. However, the printing of complex parts inevitably involves the printing of different cross-section layers. Figure 3c shows that the cones with a bottom diameter of ≈20 mm and a height of ≈30 mm were manufactured by printing the layers with gradually decreasing the diameter of circular layers. Although the surface finishing was not good enough for direct use, the cones without any delamination and the slope angle around 70° were obtained, which allowed the successful change of printing diameters of the circular cross-sections for complex geometries. Tubular PC parts are practically used for membrane reactors and fuel cells because of the easiness of assembly, which conventionally is manufactured by extrusion and casting. The rings or short tubes were prepared by 3D printing to check the capability for making thin-wall tubes or other parts, which usually has many challenges. As shown in Figure 2, the proper paste viscosity has to be satisfied with keeping the shape and obtaining good bonding and merging between layers. Figure 3d shows that the PC anode rings with ID = 15 mm, OD = 20 mm, and height 20 mm were successfully obtained by 3D printing, which proved the capability of printing thin-wall green parts. For the practical application, the one end-closed tubes usually are preferred, which can save the complicated post-sealing process or significantly simplify the design of membrane reactors. Figure 3e shows that 15 perfect bottom-closed tubes with OD of 20 mm, ID of 15 mm, and length of 40–75 mm were successfully manufactured. The green tubes with membrane area as large as 47 cm^2^ could be fabricated by 3D printing. Figure 3f shows that the tubes with smaller diameters and a continuous and round closed end at the top were also successfully manufactured by 3D printing, which in fact could be directly used as anode support for the preparation of PC half cells and single cells. Figure 3f indicates that the free-standing tubes usually have an OD of 15 mm, an ID of 10 mm, and a length up to 95 mm. One strategy for achieving a large surface area to volume ratio for PCEDs is to fabricate lobed-tube as described by the 3D model shown in Figure 3g. The lobe number and depth can be adjusted to enhance the active membrane area per unit volume. Figure 3h shows that a 4-lobed tube with a closed bottom end was successfully manufactured, which proved that the microextrusion-based 3D printing could fabricate complex geometries. Therefore, we conclude that our microextrusion-based 3D printing can successfully manufacture PC parts with versatile geometries, which not only can expand the application of PCs but also can lower the manufacturing prices or simplify the manufacturing for PCEDs. 

### 3.3. Laser Machining-Assisted 3D Printing of High-Quality PC Green Parts

It is always a dilemma for choosing fast 3D printing speed or high feature accuracy. The choice of nozzle diameter of the microextruder determines the paste filament diameter and complex structure dimension and finishing accuracy. In our L3DP technique, the laser drying, laser cutting, and laser polishing were introduced during the microextrusion-based 3D printing process to achieve high-quality complex protonic ceramic parts.

**Figure 4:**
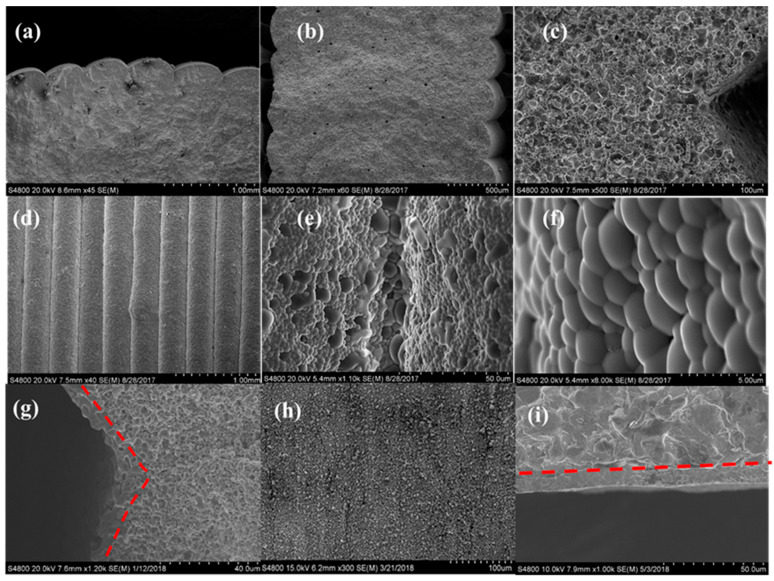
SEM characterization of green and sintered 40 wt% BZY20 + 60 wt% NiO tubes. (**a**) Cross-section of green tube, (**b**) low, and (**c**) high magnification images of the cross-sections of the sintered tube, (**d**–**f**) are the SEM images with of the surface of the sintered tubes. (**g**) Cross-section of half-cells coated on non-polished stage-surface, (**h**) the smooth surface of the laser-polished green surface, and (**i**) cross-section of the half cell with homogenous electrolyte layer coated on the anode.

In Section 3.1, we demonstrated that the low paste viscosity could not well hold the paste filament shape out of microextruder nozzle, which usually flattened and resulted in the failure of printing thin-wall protonic ceramic parts (e.g., tubes). However, the viscosity resulted in thin, smooth, and homogeneous thin films. The PC anode ring shown in Figure 1f was manufactured using the paste with relatively low viscosity followed by CO_2_ laser drying after printing each fresh layer. The laser drying quickly consolidated each printed layer, which not only allowed smooth printing (less clogging) but also resulted in excellent bonding. The SEM image (Figure 4a) of the cross-section of a 3D printed green 40 wt% BZY20 + 60 wt% NiO tube with the assistance of laser drying indicates that the interfaces between neighbor layers could not be observed, although the side view shows the existence of valleys. Furthermore, the sintered tubes were characterized by SEM, and the results are summarized in Figure 4b–f. Figure 4b shows that the homogenous layers with thicknesses of ≈340 μm were obtained. Figure 4c indicates that the neighboring layers grew together entirely without any interfacial characteristics that could be found. The SEM images of the tube surface with different magnifications further show that the sintered layers were well bonded and rounded without any sharp change observable. 

**Figure 5:**
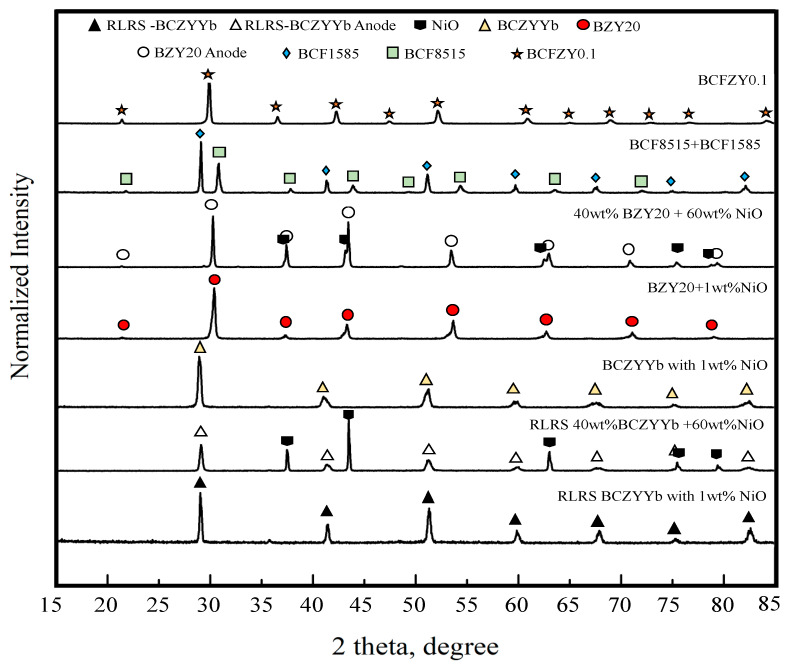
XRD patterns of the 3D printed samples after either solid state reactive sintering (SSRS) or rapid laser reactive sintering (RLRS). The samples labeled with RLRS indicate that those samples were obtained using RLRS. The samples without RLRS labels indicate that those samples were obtained using SSRS.

As we all know, the crystal structure of the sintered PC parts can directly affect the performance of PCEDs. The XRD measurements were performed on the 3D printed PC samples after SSRS in a high-temperature furnace. Figure 5 indicates that both the BZY20 + 1 wt% NiO electrolyte and the 40 wt% BZY20 + 60 wt% NiO achieved the desired crystal structures. The pure perovskite phase was obtained for electrolyte, and the mixed perovskite and nickel oxide phases were obtained for the anode. 

**Figure 6:**
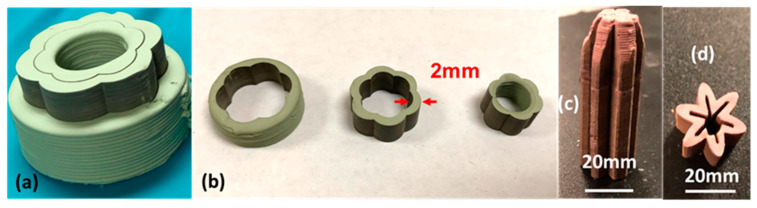
Green PC parts having more complex geometry fabricated by laser cutting assisted 3D printing. (**a**) Six-lobed 40 wt% BZY20 + 60 wt% NiO anode tube, in which the outer support body was taken off in half. (**b**) The outer support body (left), six-lobed 40 wt% BZY20 + 60 wt% NiO tube (center), and the inner support body (right). (**c**) BaCe_0.85_Fe_0.15_O_3-δ_–BaCe_0.15_Fe_0.85_O_3-δ_ tube having six high-aspect-ratio lobes, and (**d**) its cross-section.

The green PC parts with much more complicated geometries and higher demand for accuracy can be achieved by laser cutting-assisted 3D printing. The geometry of the parts was controlled not only by the paste extrusion process but also by the laser cutting. The CAD models of the protonic ceramic parts were designed to be slightly larger than their desired size on purpose. The support body of each printed layer was cut off by the ps-laser during the layer-by-layer construction. Figure 6a shows that a six-lobed short tube of anode 40 wt% BZY20 + 60 wt% NiO was manufactured by laser cutting assisted 3D printing. Its original CAD model was a cylinder without special features. Its complex geometry was made by cutting each printed layer into the six-lobed circular cross-section during the layer-by-layer construction. After removing the inner and outer supports, the short tube with six lobes on both inside and outside was successfully obtained (Figure 6b). The as-resulted inner support part was another short tube with six lobes on the outside surface and a smooth circular inside surface. The capability of laser cutting assisted 3D printing was further demonstrated by fabricating a top-closed tube with six deep outside and inside lobes (Figure 6c,d) comprised of a dual-phase hydrogen-permeable protonic membrane (BaCe_0.85_Fe_0.15_O_3-δ_-BaCe_0.15_Fe_0.85_O_3-δ_, Figure 5). 

The PC green parts usually were submitted to post-treatment, such as coating and sintering. For example, the 3D printed protonic ceramic anode green tubes were submitted to dip-coating to introduce a thin BZY20 + 1 wt% NiO electrolyte film to fabricate half cells and single cells. Figure 4g (SEM image of the cross-section of a sintered tubular half-cell) indicates that the thickness of the electrolyte film at the valley regions is thicker than the region with an outward curvature. The unevenness of the electrolyte thin film resulted from the staged surface of the green tube (Figure 4a) can cause poor current destitution during the electrochemical operation of PCEDs, which should be avoided [41]. In our L3DP system, the ps-laser was used to polish the staged surface of the 3D printed 40 wt% BZY20 + 60 wt% NiO anode tubes to get a smooth surface for performing high-quality dip-coating of BZY20 + 1 wt% NiO electrolyte thin film. Figure 4h (the SEM image of the laser-polished surface of green tube) indicates that the valleys were efficiently removed, and an excellent smoothness was obtained. Figure 4i (the SEM image of the cross-section of the sintered BZY20 + 1 wt% NiO | 40 wt% BZY20 + 60 wt% NiO tubular half-cell) indicates that the homogeneous electrolyte thin film was successfully deposited the tubular anode. No stage effect was observed. 

### 3.4. Manufacturing of PC Parts by L3DP Followed by Post-Treatment 

#### 3.4.1. Electrochemical Half Cells

**Figure 7:**
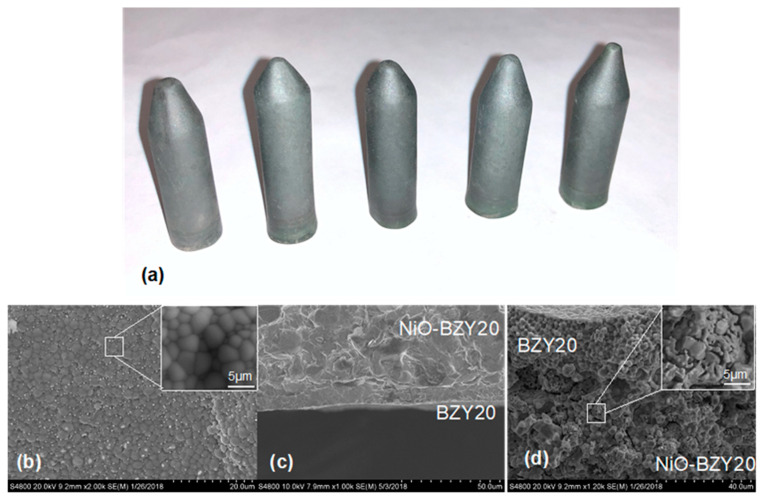
(**a**) Appearance of 40 wt% BZY20 + 60 wt% NiO | BZY20 +1 wt% NiO tubular half cells, their (**b**) surface and (**c**) cross-sectional SEM images, and (**d**) cross-sectional SEM images of the reduced samples.

The L3DP-derived PC green parts were usually subjected to coating, firing, reducing processes for being utilized as PECDs since most of them are multi-layered cells. This study demonstrated that the L3DP followed by post-treatment could be successfully utilized for the manufacturing of 40 wt% BZY20 + 60 wt% NiO | BZY20 + 1 wt% NiO tubular half cells. The 40 wt% BZY20 + 60 wt% NiO green anode tubes fabricated by the L3DP, including the laser polishing process, were prefired to remove most of the organic additives. After that, the BZY20 + 1 wt% NiO electrolyte precursor slurry was coated by dip-coating on them. The resulted green half cells were co-fired at 1550 °C for 18 h to get sintered hall cells. Figure 7a shows the appearance of the co-fired 40 wt% BZY20 + 60 wt% NiO | BZY20 + 1 wt% NiO tubular half-cells. Their surfaces have a black color with shiny reflection, which was due to the full densification of BZY20 + 1 wt% NiO electrolyte, as shown in Figure 7b. Figure 7c shows that the BZY20 + 1 wt% NiO dense layer was uniformly coated at a thickness of 15 µm. No delamination of the coated BZY20 + 1 wt% NiO electrolyte layer from the 40 wt% BZY20 + 60 wt% NiO anode support was observed, even the firing shrinkage was as high as ≈35%. The desired crystal structures for electrolyte and anode were also obtained. After the reduction treatment in a 5% H_2_ atmosphere at 600 °C, no delamination occurred between anode and electrolyte layers. (Every samples’ bonding conditions were investigated by multiple SEM images. Only the representative image was provided here.) The BZY20 + 1 wt% NiO electrolyte layer is fully dense, while ≈27% of porosity was formed for the 40 wt% BZY20 + 60 wt% Ni anode because of the reduction of NiO to Ni (Figure 7d).

#### 3.4.2. Microchannel Membranes

**Figure 8:**
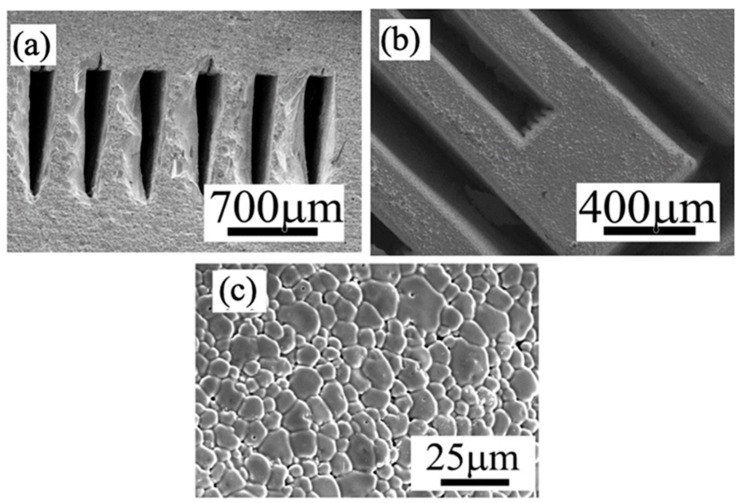
SEM images of (**a**) cross-section, (**b**) top view, and (**c**) microstructure of the microchannels embedded in the BCFZY0.1 membrane.

PC membrane reactors are facing the common significant challenge like other membrane reactors that the active membrane area per unit volume is very low for the conventional planar and tubular geometries. The novel concept of microchannel membrane reactors with a large surface-area-to-volume ratio has caught significant attention recently [35,42], which, however, has to face the manufacturing obstacles. Our L3DP method based 3D printing and laser cutting allowed the manufacturing of microchannel membrane reactors. As a case study, the triple (O^2−^, H^+^, and e^−^/h^+^) conducting PC of BCFZY0.1, which may work as oxygen or water-permeable membranes, was fabricated into a microchannel-embedded membrane using L3DP method. Figure 7 provides the SEM characterization of the microchannel BCFZY0.1 membrane prepared by the L3DP and post-sintering in a furnace at 1400 °C for 5 h. The correct crystal phase was formed with the XRD result presented in Figure 5. The microchannels were engraved by cutting each printed layer during construction; likewise, the fabrications of the objects having complex geometries. The microchannels, which were 1-mm deep and 300-µm width triangular shape and aligned in 200 µm pitch, had no shape distortion even after the firing (Figure 8a). The delamination between printed layers was not observed. Figure 8b shows the top view of the microchannels (after removing the upper part of the microchannels). No cracks in the horizontal direction were observed even at their corners. Figure 8c further indicates that the microchannels were fully densified. This demonstration suggests that the L3DP technique is capable of fabricating the PC parts with complex geometries and also having the micro-scale structures.

### 3.5. Manufacturing of PC Parts by Direct L3DP

**Figure 9:**
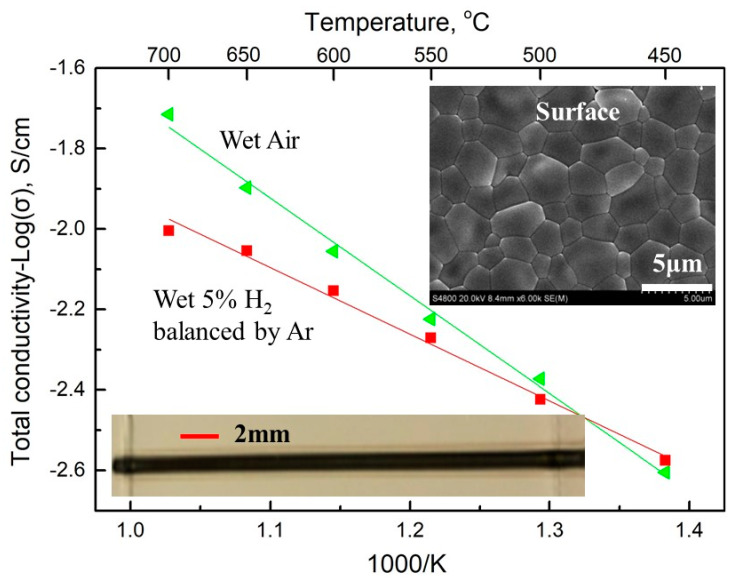
The conductivity results of the RLRS BCZYYb electrolyte sample, with the morphology of the sample and microstructure of the sample in surface and cross-section view. The size of the sintered strip is 1mm in width, 80 mm in length, and 0.16mm thick. (The black part is the sintered BCZYYb electrolyte strip, and the white area is the non-sintered green layer.)

**Figure 10:**
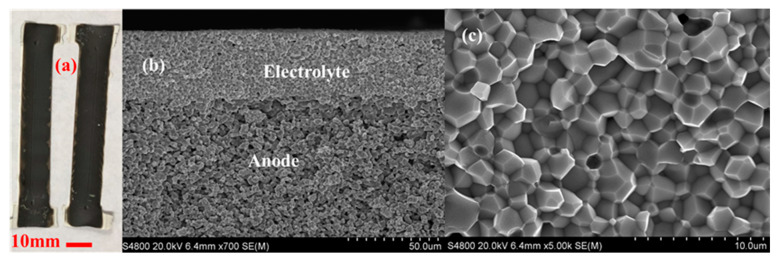
The microstructures of 40 wt% BCZYYb + 60 wt% NiO anode supported BCZYYb + 1 wt% NiO electrolyte half-cells obtained by 3D printing integrated with lasering. (**a**) Photos of two half-cells, (**b**) SEM image of the cross-section of the half-cells, and (**c**) high-magnification SEM image of the electrolyte.

The direct L3DP has been extensively used to fabricate plastic and metal parts with desired geometry, property, and function without further heavy post-treatment because the lasers were in-situ used to activate the polymerization or melt metal powders. However, the direct laser sintering of ceramics usually resulted in a lot of cracks, which has not been successfully demonstrated yet. In our new L3DP system, we demonstrated the capability to directly fabricate PC parts just out of the 3D printing stage without further complicated post-treatment. In the first step, BCZYYb with 1 wt% NiO electrolyte strip was fabricated by RLRS 3D printed green layer. The green layer was 3D printed on to the fused silica with ≈400 µm. The laser power was set at 20 W, 1 mm/s speed with 10 mm defocus distance and point shape laser lens was applied. The fully densified large-grain electrolyte film was obtained (inset photo and SEM image in Figure 9) after the RLRS. The XRD pattern (Figure 5) shows that the phase-pure BCZYYb perovskite was obtained. The proton conductivity of the BCZYYb thin film was measured by electrochemical spectroscopy at different temperatures. The sample was cut into size 5 × 1 × 0.16 mm for testing. Two atmospheres were applied, the wet argon and 5% hydrogen balanced in argon. The temperature varied from 450 to 700 °C with a step size of 50 °C. The promising proton conductivity of 6.95 × 10^−3^ S/cm was obtained at 600 °C in wet 5% H_2_ atmosphere. [33] Furthermore, the PC half cells comprised of 40 wt% BCZYYb + 60 wt% NiO anode and BCZYYb + 1 wt% NiO electrolyte was fabricated by direct 3D printing integrated with laser sintering. The 40 wt% BCZYYb + 60 wt% NiO anode precursor layer was deposited onto fused silica via 3D printing with the paste, prepared from procerus mixture of oxides and carbonates with thickness ≈300 µm. Then the BCZYYb electrolyte thin layer was coated onto the anode layer by spray coating with thickness ≈60 µm. The green half cells were then obtained by further CO_2_ laser sintering through the cylindrical lens. The one-step co-sintering of anode and electrolyte layers into a half-cell was achieved under 90 W power, 0.1 mm/s speed, and 15 mm defocus distance within several minutes. The obtained half-cell and the microstructure are shown in Figure 10. It can be clearly seen the half-cell prepared by 3D printing integrated with laser sintering (direct L3DP) has an active area larger than 10 cm^2^ (Figure 10a). The thin and fully densified electrolyte film was well bonded on the porous anode layer (Figure 10b). The high-magnification SEM image of the half-cells shows that electrolyte was fully densified. The narrow grain size distribution around 2 μm was obtained (Figure 10c). The XRD results of the RLRS-derived BCZYYb electrolyte and BCZYYb anode in the half-cell are shown in Figure 5 with RLRS BCZYYb with 1 wt% NiO and 40 wt% BCZYYb + 60 wt% NiO. The XRD results proved that the desired crystal structures were obtained for both electrolyte and anode. The performance of the cells fabricated via this technique is undergoing, which will be reported in our future publications. Therefore, we can conclude that the direct L3DP can achieve PCED half-cells with crystal structures and microstructures similar to those obtained from the SSRS method within much shorter processing time. 

## 4. Conclusions

This work developed a new technique, laser 3D printing (L3DP) method by integrating 3D and laser processing (e.g., rapid drying, rapid sintering, precise polishing, and precise cutting), that is capable of fabricating the green and sintered protonic ceramic parts for intermediate-temperature protonic ceramic devices with various complex geometries and controlled microstructures. As a demonstration, the protonic ceramic pellets, cylinders, cones, rings, straight tubes with either closed bottom or top, lobed-tube with closed bottom were successfully printed using the printable paste developed by us. The materials of NiO-BZY20 and NiO–BCZYYb anode, BZY20, and BCZYYb electrolyte, triple conducting BCFZY0.1 oxygen/water permeable membrane materials, and BCF-BFC hydrogen-permeable composite membrane materials were involved. The effectiveness of laser drying, laser cutting, laser polishing, and laser sintering was demonstrated. PC parts of the 40 wt% BZY20 + 60 wt% NiO | BZY20 + 1 wt% NiO tubular half cells, the BCFZY0.1 microchannel membrane, and the planar 40 wt% BCZYYb + 60 wt% NiO | BCZYYb + 1 wt% half cells were prepared by either L3DP with proper post-treatment (e.g., SSRS) or direct L3DP (RLRS).

## Disclaimer

This report was prepared as an account of work sponsored by an agency of the United States Government. Neither the United States Government nor any agency thereof, nor any of their employees, makes any warranty, express or implied, or assumes any legal liability or responsibility for the accuracy, completeness, or usefulness of any information, apparatus, product, or process disclosed, or represents that its use would not infringe privately owned rights. Reference herein to any specific commercial product, process, or service by trade name, trademark, manufacturer, or otherwise does not necessarily constitute or imply its endorsement, recommendation, or favoring by the United States Government or any agency thereof. The views and opinions of authors expressed herein do not necessarily state or reflect those of the United States Government or any agency thereof.
